# Lower Miocene Stratigraphy along the Panama Canal and Its Bearing on the Central American Peninsula

**DOI:** 10.1371/journal.pone.0002791

**Published:** 2008-07-30

**Authors:** Michael Xavier Kirby, Douglas S. Jones, Bruce J. MacFadden

**Affiliations:** 1 Center for Tropical Paleoecology and Archaeology, Smithsonian Tropical Research Institute, Balboa, Republic of Panama; 2 Florida Museum of Natural History, University of Florida, Gainesville, Florida, United States of America; Natural History Museum of Los Angeles County, United States of America

## Abstract

Before the formation of the Central American Isthmus, there was a Central American Peninsula. Here we show that southern Central America existed as a peninsula as early as 19 Ma, based on new lithostratigraphic, biostratigraphic and strontium chemostratigraphic analyses of the formations exposed along the Gaillard Cut of the Panama Canal. Land mammals found in the Miocene Cucaracha Formation have similar body sizes to conspecific taxa in North America, indicating that there existed a terrestrial connection with North America that allowed gene flow between populations during this time. How long did this peninsula last? The answer hinges on the outcome of a stratigraphic dispute: To wit, is the terrestrial Cucaracha Formation older or younger than the marine La Boca Formation? Previous stratigraphic studies of the Panama Canal Basin have suggested that the Cucaracha Formation lies stratigraphically between the shallow-marine Culebra Formation and the shallow-to-upper-bathyal La Boca Formation, the latter containing the Emperador Limestone. If the La Boca Formation is younger than the Cucaracha Formation, as many think, then the peninsula was short-lived (1–2 m.y.), having been submerged in part by the transgression represented by the overlying La Boca Formation. On the other hand, our data support the view that the La Boca Formation is older than the Cucaracha Formation. Strontium dating shows that the La Boca Formation is older (23.07 to 20.62 Ma) than both the Culebra (19.83–19.12 Ma) and Cucaracha (Hemingfordian to Barstovian North American Land Mammal Ages; 19–14 Ma) formations. The Emperador Limestone is also older (21.24–20.99 Ma) than the Culebra and Cucaracha formations. What has been called the “La Boca Formation” (with the Emperador Limestone), is re-interpreted here as being the lower part of the Culebra Formation. Our new data sets demonstrate that the main axis of the volcanic arc in southern Central America more than likely existed as a peninsula connected to northern Central America and North America for much of the Miocene, which has profound implications for our understanding of the tectonic, climatic, oceanographic and biogeographic history related to the formation of the Isthmus of Panama.

## Introduction

The paleogeography of Central America has changed profoundly over the past 30 million years (m.y.), from a volcanic arc separated from South America by a wide seaway, to an isthmus that connected North and South America by 3 Ma [1]–[5]. The formation of the Isthmus of Panama was important because it allowed the mixing of terrestrial faunas between the two continents [6], as well as physically separating a once continuous marine province into separate and distinct Pacific and Caribbean communities [7]–[12]. The formation of the Isthmus of Panama also ultimately led to profound changes in global climate [13] by strengthening the Gulf Stream and thermohaline downwelling in the North Atlantic [14]–[17].

**Figure 1:**
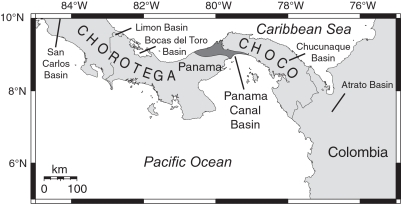
Location of the Panama Canal Basin and other depositional basins in southern Central America.

Although extensive study has constrained the timing of isthmian formation [1]–[4], [18]–[20], the paleogeographic nature of southern Central America before the isthmus is still disputed. Paleobathymetric and other geologic evidence from depositional basins suggests that southern Central America arose slowly from bathyal depths during the Neogene as a result of the collision between the Panama microplate and the South American plate [3], [4], [21], suggesting that the volcanic arc during the Miocene consisted of an archipelago of volcanic islands that was slowly uplifting through the Neogene until the ultimate formation of the isthmus [2]–[5], [22]. For example, Coates et al. [2] stated that (p. 816): “It is likely that during the late Neogene the Chorotega and Choco blocks formed an archipelago and there were frequent marine connections between the Caribbean and the Pacific (Duque-Caro, 1990b, his Figure 7). The topographic, tectonic, and regional geologic evidence strongly suggests that the archipelago stretched from westernmost Costa Rica to the Atrato Valley in Colombia …” However, most of the evidence suggesting slow uplift of the volcanic arc from bathyal depths is derived from depositional basins that lie peripheral to the main axis of the volcanic arc in southern Central America (Figure 1).

An alternative view is that the main axis of the volcanic arc had already arisen above sea level by the early Neogene, which would effectively make Panama a peninsula of Central America by this time [23]. Evidence supporting this latter view comes from land mammal fossils found in the Miocene Cucaracha Formation exposed in the Gaillard Cut of the Panama Canal near the center of the Panama Canal Basin (Table 1). Land mammals with only North American affinities and similar body sizes to conspecific taxa in North America suggest a terrestrial connection with North America by the early Miocene [23]–[26]. The purpose of the present study is to further resolve the Neogene paleogeography of southern Central America by placing the Cucaracha land mammals into a stratigraphic framework through lithostratigraphic, biostratigraphic and strontium chemostratigraphic analyses that test long-standing hypotheses concerning the stratigraphy of the Gaillard Cut.

**Table 1:** Land mammal taxa from the Gaillard Cut Local Fauna, Cucaracha Formation, Panama.

Order	Family	Genus & species	Common name	Biogeographic affinity
Rodentia	–	*Texomys stewarti*	Geomyoid rodent	North America
Carnivora	Canidae	*Tomarctus brevirostris*	Dog	North America
Carnivora	Amphicyonidae or Hemicyonidae	*–*	Bear dog	North America
Artiodactyla	Tayassuidae	cf. *Cynorca* sp.	Peccary	North America
Artiodactyla	Oreodontidae	*Merycochoerus matthewi*	Oreodont	North America
Artiodactyla	Protoceratidae	*Paratoceras wardi*	Protoceratid	North America
Perissodactyla	Equidae	*Anchitherium clarencei*	Horse	North America
Perissodactyla	Equidae	*Archaeohippus* sp.	Horse	North America
Perissodactyla	Rhinocerotidae	*Menoceras barbouri*	Rhinoceros	North America
Perissodactyla	Rhinocerotidae	*Floridaceras whitei*	Rhinoceros	North America

Source: [23], [25], [26], [71]

**Figure 2:**
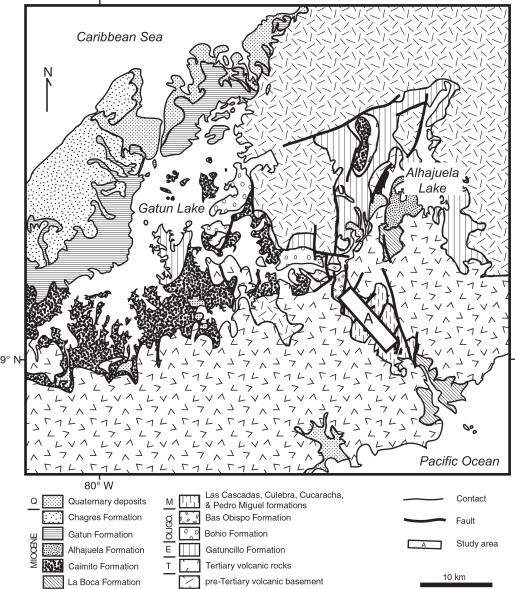
Geologic map of the Panama Canal Basin showing the study area (A) in relation to rock units discussed in the text. Geology modified from Stewart et al. [27]. Q = Quaternary. M = Miocene. Oligo. = Oligocene. E = Eocene. T = Tertiary.

**Figure 3:**
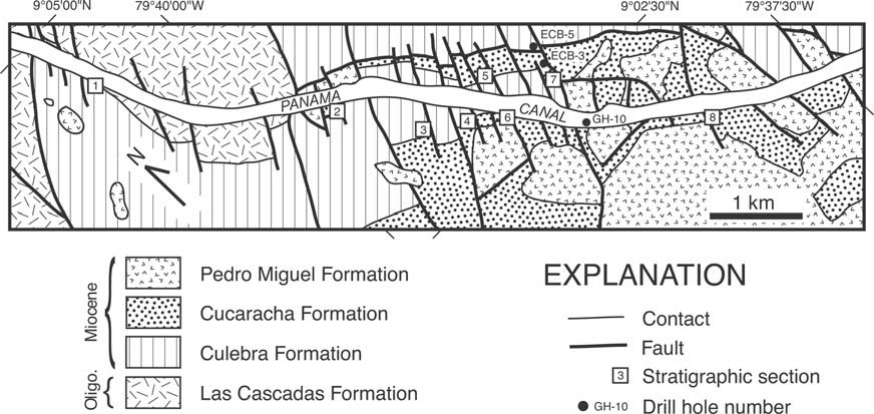
Geologic map of the study area along the Gaillard Cut portion of the Panama Canal, within the Panama Canal Basin (see rectangle labeled ‘A’ in Figure 2). Geology modified from Stewart et al. [27]. Oligo. = Oligocene.

Excavation of the Gaillard Cut during the original construction of the Panama Canal exposed lower Neogene sediments of the Panama Canal Basin (Figures 2 and 3). As upper Neogene volcanic rocks cover much of the Panama Canal Basin, the Gaillard Cut offers a window into the underlying Oligocene-Miocene rocks beneath this volcanic cover (Figure 2). Although this excavation exposed hundreds of meters of section, the structural complexity caused by extensive faulting has obscured the stratigraphic relationships between the various formations, such that only a portion of one, or at most two, formations are present in any given fault-bounded block (Figure 3). The most recently published stratigraphy and geologic map for the Panama Canal Basin indicates that the Cucaracha Formation lies stratigraphically above the shallow-marine Culebra Formation and below the shallow-to-upper-bathyal La Boca Formation [27]. If this stratigraphic arrangement is correct, then we may conclude that the peninsula containing North American land mammals was short-lived in the early Neogene (1–2 m.y., based on the temporal duration of paleosols in the Cucaracha Formation [28]), having been submerged in part by the marine transgression represented by the overlying La Boca Formation [23]. Earlier stratigraphic arrangements, however, placed strata presently in the La Boca Formation not above the Cucaracha Formation, but below it [29]–[31]). Given this stratigraphic arrangement, the marine transgression represented by the La Boca Formation occurred before deposition of the Cucaracha Formation, which would indicate that there is no evidence for submergence of the Central American Peninsula until 6 Ma, when there is evidence for a short-lived strait across the Panama Canal Basin [22]. Was the Central American Peninsula short-lived, existing for only the 1 to 2 million years that it took to form the Cucaracha Formation? Or did the peninsula exist longer than this? Although we cannot currently determine exactly when the Central American Peninsula formed or how far east it may have extended, we can constrain the interval of time that such a peninsula may have existed by placing the land mammals of the Cucaracha Formation into a well-defined stratigraphy. Lithostratigraphic, biostratigraphic and strontium chemostratigraphic analyses presented here allow us to test the validity of different stratigraphic models proposed for the Gaillard Cut. Correlation from lithostratigraphic analysis of 11 stratigraphic sections, biostratigraphic placement of the fossil land mammals and absolute age estimates from strontium isotopes of marine fossils are used for the first time to test the different stratigraphic models that have been proposed for the Gaillard Cut. The solution to these paleogeographic and stratigraphic problems has profound implications for our understanding of the biogeographic, paleoclimatic, tectonic and evolutionary history of the Isthmus of Panama. Furthermore, many studies rely on a proper understanding of the stratigraphy of the Gaillard Cut [25], [28], [32]. The resolution of this stratigraphic problem also has important application to geotechnical studies associated with the expansion of the Panama Canal that is planned to occur between 2008 and 2014.

### Regional geologic setting

The Panama Canal Basin is a Tertiary structural and depositional basin that straddles the tectonic boundary between the Chorotega and Choco blocks of the Panama microplate (Figure 1) [5], [33]. As part of the Central American volcanic arc, the Panama microplate formed through subduction of various oceanic plates during the Cretaceous and Cenozoic [34]. This microplate lies between the Cocos and Nazca plates to the south, the Caribbean plate to the north and the South American plate to the east [34]. The formation of the Panama Canal Basin may be related to the hypothetical “Gatun Fault Zone,” which may represent the tectonic boundary between the Chorotega and Choco blocks [5], [35]. Case [35] inferred the existence of a deep-shear zone trending northwest-southeast, approximately parallel to the Panama Canal, based on gravity data indicating a very steep gradient underlying the Panama Canal Basin. He speculated that this concealed fault zone may have had lateral displacement and could be of early Cenozoic or older age. Lowrie et al. [36] also recognized a major fault zone in this area based on several lines of evidence. Later studies, however, have suggested that there is no direct evidence for the existence of the Gatun Fault Zone [37]. Nevertheless, the Panama Canal Basin exists in a structurally complex area, as indicated by thousands of northeast-southwest trending sets of faults [37]. The exact tectonic nature of this basin continues to remain unclear, but it may represent an active rift or forearc basin [34].

The Panama Canal Basin contains a thick sequence of sediments and volcanic rocks (>2900 m) of Eocene to Pleistocene age (Figure 2) [27], [38]–[41]. The lowermost sedimentary unit is the Eocene Gatuncillo Formation, which contains marine mudstone, siltstone and limestone, and unconformably overlies pre-Tertiary volcanic basement [40], [41]. Overlying the Gatuncillo Formation is the Oligocene Bohio Formation, which contains marine and non-marine conglomerate, tuffaceous sandstone and siltstone [40], [41]. Stratigraphically higher are the Oligocene Bas Obispo and Las Cascadas formations, both of which consist of agglomerate and tuff [40]. Conformably overlying the Las Cascadas Formation is the lower Miocene Culebra Formation (this study), which contains marine mudstone, sandstone, limestone, conglomerate and lignite. Portions of the La Boca, Alhajuela and Caimito formations are correlative with the Culebra Formation, as suggested by this study and previous lithostratigraphic and biostratigraphic studies [30], [31], [38], [40]. The lower to middle Miocene Cucaracha Formation overlies the Culebra Formation and consists of subaerial claystone, sandstone, conglomerate and lignite, all showing paleosol development [28]. The middle Miocene Pedro Miguel Formation overlies conformably the Cucaracha Formation and contains basalt and agglomerate. Stratigraphically higher is the upper Miocene Gatun Formation [33], which contains marine siltstone, sandstone and conglomerate [22], [41]. East of the city of Colon, the Gatun Formation overlies nonconformably unnamed Cretaceous volcanic rocks; whereas, west of Colon, the Gatun Formation overlies unconformably the Caimito Formation [33]. The Gatun Formation is overlain disconformably by the upper Miocene Chagres Formation [33], which consists of conglomeratic sandstone and a basal coquina of the Toro Member [22]. Unconformably above the Tertiary formations are unconsolidated Quaternary deposits, informally known as the “Pacific muck” and “Atlantic muck” [40].

### Stratigraphic models for the Gaillard Cut

**Figure 4:**
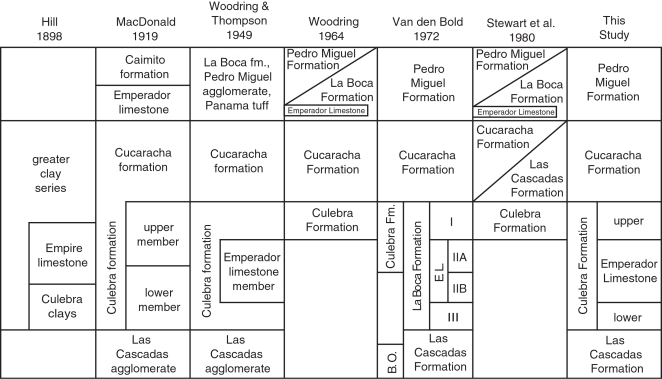
Summary of stratigraphic nomenclature for the formations exposed along the Gaillard Cut portion of the Panama Canal. B.O. = Bas Obispo Formation. E.L. = Emperador Limestone.

Hill [42] was the first to systematically name and describe formations along the Gaillard Cut (Figure 4). MacDonald [29], [43] later named and described several formations in the Panama Canal Basin, including the Las Cascadas and Cucaracha formations. Woodring and Thompson [30] formally named and described the Pedro Miguel and La Boca formations. They also placed the Emperador Limestone Member within the Culebra Formation. Based on field work by R. H. Stewart of the Panama Canal Company, Woodring [44] later restricted the Culebra Formation by placing sections containing the Emperador Limestone (that is, the lower two-thirds of the Culebra Formation) into the La Boca Formation, which he considered younger than the Cucaracha Formation. He kept the upper one-third of the Culebra Formation stratigraphically below the Cucaracha Formation. Woodring [44] did not state explicitly the reasons or evidence for this revision in the stratigraphy of the Gaillard Cut, only that the new evidence was derived from drill cores made by R. H. Stewart. Writing in 1964, Woodring [44,] stated that (p. 244): “After the drilling along the Empire Reach … got under way, R. H. Stewart, geologist of the Panama Canal, soon realized that the geology of the northwestern part of the Gaillard Cut area had been misinterpreted.” Stewart et al. [27] later hypothesized interfingering relationships between the Cucaracha and Las Cascadas formations, as well as between the Pedro Miguel and La Boca formations, in order to justify placement of sections containing the Emperador Limestone within the La Boca Formation (Figure 4) [45]. However, these interfingering relationships are not apparent in outcrop exposures or in subsurface well logs. Van den Bold [31], a noted biostratigrapher of Caribbean ostracodes, disagreed with the revised stratigraphic interpretation of Woodring [44] by demonstrating that stratigraphic sections containing the Emperador Limestone and overlying sediments (i.e. La Boca Formation) were correlative with the Culebra Formation, based on ostracode biostratigraphy. Specifically, his zones I and IIA of the “La Boca Formation” are correlative with the Culebra Formation (Figure 4). Later studies have followed the stratigraphy as originally proposed by Woodring and Thompson [30], based on data gathered in support of the current study [28], [32].

**Figure 5:**
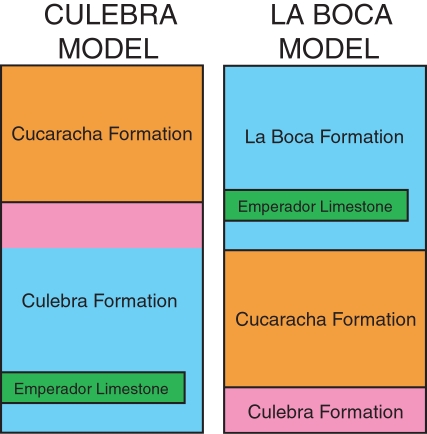
The two alternative stratigraphic models for the formations exposed along the Gaillard Cut portion of the Panama Canal: (1) the Culebra model [30], [31], [41] and (2) the La Boca model [27], [44], [45]. Equal color between the two models refer to the same stratigraphic unit.

All of the stratigraphic models that have been proposed for the Gaillard Cut over the past one hundred years can be divided into two groups, which are herein called the (1) Culebra model and the (2) La Boca model (Figure 5). The Culebra model places all of the Culebra Formation [sensu 30] underneath the Cucaracha Formation [29]–[31], [41]. The La Boca model, on the other hand, places the lower portion of the Culebra Formation [sensu 30] (marked by blue in Figure 5) into the La Boca Formation above the Cucaracha Formation [27], [44], [45]. As the upper portion of the Culebra Formation (marked by purple in Figure 5) remains beneath the Cucaracha Formation in this model, the La Boca model considers the Culebra Formation of Woodring and Thompson [30] to actually be two different formations (i.e. Culebra and La Boca formations), with the Cucaracha Formation lying between the two (Figure 5). If the La Boca Formation (marked by blue in Figure 5) is found to be younger than the Cucaracha Formation, then the Culebra model will be rejected and the La Boca model supported. Alternatively, if the La Boca Formation is found to be older than the Cucaracha Formation, then the La Boca model will be rejected and the Culebra model supported.

## Methods

**Figure 6:**
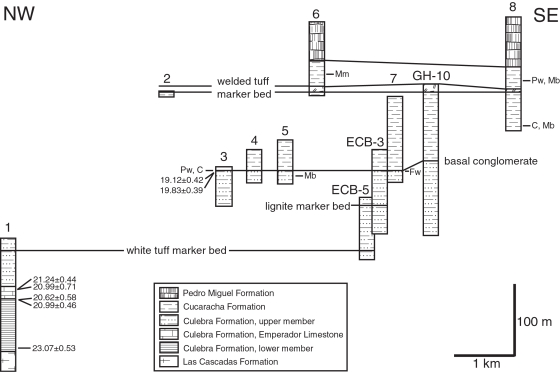
Lithostratigraphic correlation of stratigraphic sections 1 to 8 and drill cores logged by R. H. Stewart of the Panama Canal Company (ECB-5, ECB-3 and GH-10) arranged northwest to southeast along the Gaillard Cut portion of the Panama Canal. Location of stratigraphic sections and drill holes in Figure 3. Absolute ages (Ma) are derived from Sr chemostratigraphic analyses (see text). C = cf. *Cynorca* sp. Mm = *Merycochoerus matthewi*. Pw = *Paratoceras wardi*. Mb = *Menoceras barbouri*. Fw = *Floridaceras whitei*.

Field work was conducted in February 2003, July 2003 to December 2004 and March 2005. This study benefited greatly from many newly exposed surface sections made by recent widening of the Panama Canal by the Panama Canal Authority (ACP) and the construction of a second bridge across the canal (Centennial Bridge). We measured eight stratigraphic sections between the towns of Pedro Miguel and Gamboa along the Gaillard Cut portion of the Panama Canal (Figure 6). These outcrop sections were measured with a Jacob Staff and Brunton compass or with a tape and Brunton compass (methods described in Compton [46]). We collected rock and fossil samples, recording their stratigraphic position and deposited them at the Center for Tropical Paleoecology and Archaeology, Smithsonian Tropical Research Institute, and at the Florida Museum of Natural History. Well logs derived from drill cores from the archives at the ACP were also examined in order to aid correlation of surface sections and to fill in missing intervals (ECB-3, ECB-5 and GH-10 well logs at the ACP; Figure 6). We used the biochronology of the land mammals from the Gaillard Cut local fauna, as modified by MacFaddden [26]. The land-mammal chronology and North American Land Mammal Age subdivsions follow Tedford et al. [47].

Many studies have shown that the Neogene is generally a time of rapidly increasing ^87^Sr/^86^Sr in the global ocean and, hence, particularly amenable to dating and correlating marine sediments using strontium isotopes [48]–[56]. We analyzed seven fossil specimens from the restricted, upper Culebra Formation and the La Boca Formation in the Gaillard Cut, along the Panama Canal, in order to determine the ratio of ^87^Sr/^86^Sr of the calcium carbonate composing the shell (Table 2). These data allow us to estimate the geologic age for each fossil specimen. For isotopic analyses, we first ground off a portion of the surface layer of each shell specimen to reduce possible contamination. Areas showing chalkiness or other signs of diagenetic alteration were avoided. Powdered aragonite (coral) or low-magnesium calcite (mollusc) samples were drilled from the interior of each specimen using a hand-held Dremel tool with a carbide burr. Approximately 0.01 to 0.03 g of powder was recovered from each fossil sample. The powdered samples were dissolved in 100 µl of 3.5 N HNO_3_ and then loaded onto cation exchange columns packed with strontium-selective crown ether resin (Eichrom Technologies, Inc.) to separate Sr from other ions [57]. Sr isotope analyses were performed on a Micromass Sector 54 Thermal Ionization Mass Spectrometer equipped with seven Faraday collectors and one Daly detector in the Department of Geological Sciences at the University of Florida. Sr was loaded onto oxidized tungsten single filaments and run in triple collector dynamic mode. Data were acquired at a beam intensity of about 1.5 V for ^88^Sr, with corrections for instrumental discrimination made assuming ^86^Sr/^88^Sr = 0.1194. Errors in measured ^87^Sr/^86^Sr are better than ±0.00002 (2σ), based on long-term reproducibility of NIST 987 (^87^Sr/^86^Sr = 0.71024). Age estimates were determined using the Miocene portion of Look-Up Table Version 4:08/03 associated with the strontium isotopic age model of McArthur et al. [56].

**Table 2:** Sr chemostratigraphic analyses of the Cuelbra Formation, Panama.

Sample	Taxon	Latitude	Longitude	Member	^87^Sr/^86^Sr	Std. error %	Std. error (external)	Age estimate (Ma)	Std. error (Ma)
Pan8	Coral	9°04.661′N	79°40.627′W	Emperador	0.708386	0.001	0.000023	20.99	0.71
Pan9	Coral	9°04.661′N	79°40.627′W	Emperador	0.708371	0.0008	0.000023	21.24	0.44
Pan6	Pectinid	9°04.661′N	79°40.627′W	Lower	0.708404	0.0008	0.000023	20.62	0.58
Pan7	Pectinid	9°04.661′N	79°40.627′W	Lower	0.708386	0.0008	0.000023	20.99	0.46
Pan10	Bivalve	9°04.468′N	79°40.522′W	Lower	0.70825	0.0008	0.000023	23.07	0.53
Pan4	*Ostrea* sp.	9°03.099′N	79°39.350′W	Upper	0.708502	0.0008	0.000023	19.12	0.42
Pan5	Pectinid	9°03.099′N	79°39.350′W	Upper	0.70845	0.0007	0.000023	19.83	0.39

## Results

Results from our lithostratigraphic, biostratigraphic and Sr chemostratigraphic analyses of the formations exposed along the Gaillard Cut allow us to reject the La Boca model. Instead, all data sets support the Culebra model as being the correct interpretation for the stratigraphy of the formations along the Gaillard Cut (Figures 6–8). We found no evidence for interfingering relationships between the Cucaracha and Las Cascadas formations, or between the La Boca and Pedro Miguel formations, as proposed by Stewart et al. [27] and Graham et al. [45]. We did find that what has been called the La Boca Formation (i.e., lower Culebra Formation), conformably overlies the Las Cascadas Formation and that the upper Culebra Formation underlies the Cucaracha Formation, which in turn is overlain by the Pedro Miguel Formation (Figure 6).

**Figure 7 pone-0002791-g007:**
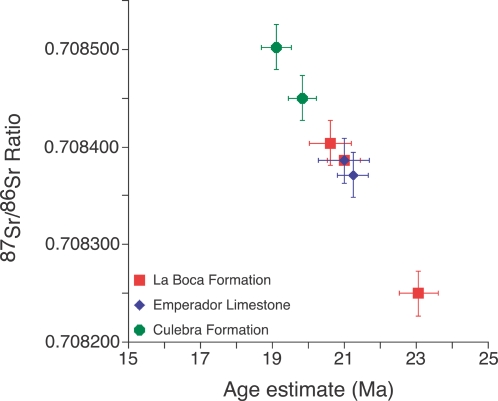
Scatter plot showing geologic age as a function of the ^87^Sr/^86^Sr ratio of fossil marine shells from the La Boca Formation, Emperador Limestone and upper Culebra Formation. Error bars represent standard error.

**Figure 8:**
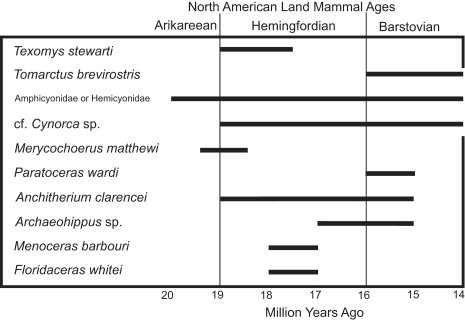
Biostratigraphy of the ten taxa of land mammals found in the Gaillard Cut Local Fauna in the Culebra and Cucaracha formations, Panama, based on the North American Land Mammal Age of their conspecific taxa in North America.

Sr analyses show that the La Boca Formation measured at Section 1 is significantly older than the uppermost Culebra Formation (23.07–20.99 Ma versus 19.83–19.12 Ma) (Figure 7). If the La Boca Formation was stratigraphically higher than the uppermost Culebra Formation, then the Sr data should have indicated a younger age for the La Boca Formation. Furthermore, the upper Culebra and Cucaracha formations contain land mammal fossils that are late Hemingfordian to Barstovian in age (19.5 to 14 Ma), which is significantly younger than the La Boca Formation measured at Section 1 (23.07–20.99 Ma) (Figure 8). Other biostratigraphic data from benthic foraminifera, ostracodes, corals and molluscs are consistent with an older, early Miocene age for the La Boca Formation [31], [32], [40], [58].

As our three data sets indicate that the Culebra model is the correct stratigraphic model for the formations exposed along the Gaillard Cut, we return the La Boca Formation containing the Emperador Limestone back to the lower Culebra Formation. This stratigraphic arrangement is consistent with that originally proposed by Woodring and Thompson [30] (Figure 4). Our lithostratigraphic, biostratigraphic and Sr chemostratigraphic results are discussed in greater detail below.

### Culebra Formation

#### Lithostratigraphy

**Figure 9:**
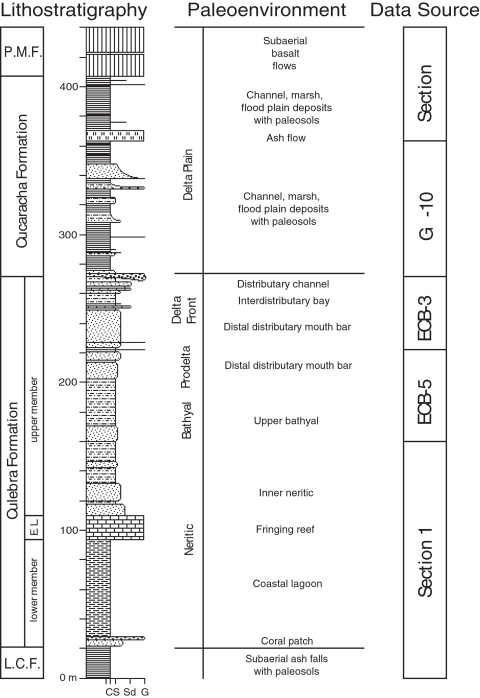
Composite stratigraphic section of the formations along the Gaillard Cut, Panama Canal Basin, showing lithostratigraphy and palaeoenvironmental interpretations, based on stratigraphic relationships illustrated in Figure 6. “Data Source” indicates the stratigraphic sections used to compile the composite section. L.C.F. = Las Cascadas Formation. E.L. = Emperador Limestone. P.M.F. = Pedro Miguel Formation. Abbreviations at the base of the section represent grain size (C = Clay; S = Silt; Sd = Sand; G = Gravel).

The Culebra Formation is at least 250 m thick and consists of three members that include a lower unnamed member, the Emperador Limestone and an upper unnamed member (Figure 9). The Culebra Formation contains a transgressive-regressive facies pattern, where the lower member, the Emperador Limestone and the lower part of the upper member show a transgressive pattern in which water depth deepened from intertidal at the base of the formation to upper bathyal [58]. The upper part of the upper member shows a regressive pattern in which water depth shallowed from upper bathyal to intertidal depths. These three members are described individually below.

The lower member of the Culebra Formation is only exposed in Section 1 (Figure 6), where it conformably overlies the Las Cascadas Formation and conformably underlies the Emperador Limestone. The Las Cascadas Formation consists of agglomerate with tuffaceous claystone interbeds representing subaerial volcanism with periods of paleosol development. The lower member of the Culebra Formation consists mostly of carbonaceous mudstone with thin tabular interbeds of fossiliferous lithic wacke. The base of the lower member is defined by a black lignitic mudstone bed that is overlain by calcarenite (calcareous sandstone) and pebble calcirudite (calcareous conglomerate). The top of the lower member is defined by a very distinctive bed of fine-grained, lithic wacke that contains pectinids (*Lepidopecten proterus*) and spondylids (*Spondylus scotti*). The lower member of the Culebra Formation represents the beginning of a transgressive sequence, with paleosols developed in volcanic sediments of the Las Cascadas Formation below the base of the Culebra Formation, and shallow-marine facies in the Culebra Formation (Figure 9). Carbonaceous mudstone and lignitic interbeds represent a shallow lagoon protected from the open ocean by a fringing reef (Emperador Limestone), which is consistent with carbonized compressions of sea grass and wood. This interpretation is also consistent with the presence of brackish *Elphidium* foraminifera and ostracodes in the lower member described by Blacut and Kleinpell [58] and Van den Bold [31], respectively. The presence of *Elphidium* and the absence of globigerinid foraminifera (the latter are common in siltstone in the upper member of the Culebra Formation) suggest a current-protected, nearshore environment [58]. The lithic wacke interbeds represent storm deposits in the lagoon. Burrows in the underlying mudstone were rapidly infilled by sand during the storm events, thereby preserving the trace fossil *Thalassinoides* sp. The lowermost calcarenite and calcirudite beds within the carbonaceous mudstone represent bioclastic debris likely derived from patches of coral in the lagoon [32].

The Emperador Limestone is the middle member of the Culebra Formation and consists of five distinct facies in Section 1 [32]. The base of the Emperador Limestone, which overlies conformably the “pectinid-spondylid” sandstone bed in the lower member of the Culebra Formation, is defined by a branching-coral boundstone containing abundant *Acropora saludensis* and *Montastraea canalis* in a very fine-grained calcarenite matrix. The second facies consists of white, rhodolithic limestone. The third facies consists of another branching-coral boundstone dominated by *Acropora saludensis, Stylophora granulata* and *Porites douvillei* as part of a diverse assemblage of corals in a mud matrix with isolated coral heads of *Montastraea imperatoris* in life position. The fourth facies consists of platy-coral boundstone. The top of the Emperador Limestone is defined by the fifth facies, which consists of calcirudite containing fragmented corals in a calcarenite matrix and displaced head corals of *Montastraea* species and massive *Porites* species. The Emperador Limestone represents a fringing reef that protected a neighboring lagoon, as represented by the carbonaceous mudstone facies in the underlying lower member [32]. The Emperador Limestone is overlain conformably by alternating beds of sandstone and siltstone of the upper member of the Culebra Formation.

The upper member of the Culebra Formation consists of five distinct facies and is best exposed in Sections 1, 3–5 and 7 (Figure 6). The base is defined by alternating beds of sandstone and siltstone that conformably overlie the Emperador Limestone in Section 1. The lowermost sandstone bed contains displaced corals and abundant molluscs. Massive *Porites* species and head corals of *Montastraea canalis* are clearly not *in situ*, as they show different orientations with respect to bedding. The sandstone beds become thinner and finer upsection, whereas the interbedded siltstone beds thicken. In addition, the sandstone beds grade upsection from a medium-grained calcarenite to a fine-grained, lithic wacke, such that carbonate grains decrease in abundance, whereas quartz and lithic grains increase in abundance. There is also a lateral facies change between these two facies, such that the sandstone beds become thinner and the siltstone beds thicken to the north over 1 km of exposure. The siltstone beds contain abundant foraminifera, molluscs, echinoids, shark teeth and burrows. A distinctive white ash bed (4 cm thick) is present at the base of a tuffaceous sandstone bed (1 m thick). This ash bed and overlying tuffaceous sandstone, which serves as a useful marker bed (Figure 6), is overlain by a thick interval of siltstone (∼30 m). Overlying this thick interval of siltstone is the uppermost interval of the upper member, which consists of alternating sandstone and mudstone beds with local conglomerate and lignite beds (Sections 3–5, 7, ECB-3, ECB-5 and GH-10). The sandstone beds become thicker and coarser upsection, whereas the mudstone beds become thinner and more carbonaceous upsection. A distinctive lignite bed is present in drill holes ECB-3 and ECB-5, which serves as a useful marker bed (Figure 6). Carbonaceous mudstone beds commonly contain horizons of carbonized to permineralized wood of mangrove trees. Branches and prop roots are commonly encrusted with oysters (*Crassostrea* aff. *C. virginica*) and are commonly bioeroded by teredinid bivalves (*Kuphus* “*incrassatus*”). Also common in the carbonaceous mudstone are poorly preserved seeds, gastropods (*Turritella venezuelana*, *Turritella* (*Bactrospira*?) *amaras*, *Potamides suprasulcatus*), bivalves and crustaceans. The top of the Culebra Formation is defined by the incision into either carbonaceous mudstone or sandstone (depending upon location) of a channel infilled with pebble conglomerate containing wood without teredinid borings of the Cucaracha Formation.

The lower half of the upper member represents continuing transgression through time; whereas, the upper half represents the start of a regression with shallowing water depth through time (Figure 9). The lowermost calcarenite beds above the Emperador Limestone represent open-shelf, neritic conditions. The decrease in bed thickness, in grain size and in carbonate content of these sandstone beds, as well as the thickening siltstone beds upsection, indicate increasing water depth through time. This interpretation is consistent with Blacut and Kleinpell [58], who described benthic foraminifera representing upper bathyal depths (∼200–400 m) from these siltstone beds. This interpretation is also consistent with Van den Bold [31], who described ostracodes representing “deep-water” depths. During deposition of the middle portion of the upper member, water depth began to shallow, with sandstone beds increasing in frequency, thickening and coarsening, and mudstone beds becoming thinner and increasing in carbonaceous plant content. All of these observations suggest a small prograding, river-dominated delta [59], [60], perhaps on a similar scale to the present-day Rio Grande in Bocas del Toro, Panama. The thick siltstone interval represents the prodelta, whereas the overlying sequence of alternating mudstone and sandstone beds represent the delta front (Figure 9). Sandstone interbeds represent distal distributary mouth bars. Local lenses of pebble conglomerate represent distributary-channel deposits. Carbonaceous mudstone beds represent interdistributary bay deposits with mangal associations of mangrove and oyster.

#### Land Mammal Biostratigraphy

Traces of land mammals are extremely rare in the Culebra Formation. We found three molars representing three taxa in the uppermost part of the Culebra Formation in the course of our work. These discoveries are the first definite report of land mammal fossils from the marine Culebra Formation (although see Woodring [40], who reported a partial ungulate metapodial from what he described as the transition zone between the Culebra and Cucaracha formations). We found molars of the artiodactyl *Paratoceras wardi* and peccary cf. *Cynorca* sp. at the top of the Culebra Formation in Section 3 (Figure 6), as well as a molar of the rhinoceros *Menoceras barbouri* in the uppermost part of the Culebra Formation in Section 5 (Figure 6). Based on their respective ages in North America, *Paratoceras wardi* is Barstovian (16–15 Ma), cf. *Cynorca* sp. is probably early Hemingfordian to Barstovian (18.8–14 Ma) and *Menoceras barbouri* is Hemingfordian (18–17 Ma) (Figure 8) [26], [61]. Taken together, these fossils suggest an age of between 18.8 and 14 Ma for the uppermost part of the Culebra Formation.

#### Strontium Chemostratigraphy

In order to derive age estimates for the Culebra and La Boca formations, we collected seven samples of fossil coral and bivalves from two sections containing the La Boca and upper Culebra formations and analyzed them for their ^87^Sr/^86^Sr ratios (Figure 7; Table 2). A fossil bivalve-shell fragment from the calcirudite bed near the base of the La Boca Formation in Section 1 (i.e., the lower member of the Culebra Formation) had an estimated age of 23.07±0.53 Ma, based on its ^87^Sr/^86^Sr ratio. Two pectinid bivalves from the “pectinid-spondylid” sandstone bed below the overlying Emperador Limestone had an estimated age of 20.62±0.58 and 20.99±0.46. Two *Acropora* coral specimens from the upper branching facies of the Emperador Limestone had an estimated age of 20.99±0.71 and 21.24±0.44. In Section 3, two pectinid bivalves collected from a fine-grained calcarenite bed (2 m below the conglomeratic sandstone containing *Paratoceras wardi* and cf. *Cynorca* sp. specimens) of the upper member of the Culebra Formation had an estimated age of 19.12±0.42 and 19.83±0.39. Taken together, the samples from the La Boca Formation are one to four million years older than the samples from the upper Culebra Formation (Figure 7; Table 2).

### Cucaracha Formation

#### Lithostratigraphy

The Cucaracha Formation is about 140 m thick and consists mostly of claystone with a minor amount of conglomerate, sandstone, lignite and welded tuff (Figure 9). Lenticular beds of conglomerate and sandstone are more common in the lower half of the formation below a distinctive welded tuff bed of volcanic origin, whereas tabular beds of claystone and lignite are more common in the upper half above the welded tuff bed (more specifically, the lower half of the formation has a sandstone/claystone ratio of 24.1%; whereas, the upper half has a sandstone/claystone ratio of only 5.7%). The base of the Cucaracha Formation is marked by a distinctive pebble conglomerate bed that lies unconformably over the Culebra Formation (Figure 6). This conglomerate bed is widely distributed and contains volcanic pebble clasts with rare fragments of carbonized wood (without teredinid borings) and oysters. This and other pebble conglomerate beds higher up in the Cucaracha Formation commonly become finer upsection, grading into lithic wacke, siltstone and claystone. Medium to coarse-grained, lithic wacke beds are commonly cross-bedded, which show an average paleocurrent direction to the east (N87°E±6.4° [95% confidence cone, Fisher analysis]). These interbedded channel deposits contain permineralized logs of up to 1 m in length and 30 cm in diameter oriented parallel to bedding. Pebble conglomerate and lithic wacke also contain rare fossils of land mammals. Olive-gray to blackish red claystone is the most common lithology in the Cucaracha Formation. This claystone is commonly structureless to slickensided, but may contain mottling and drab-haloed root traces. Horizons of calcite nodules and rhizoconcretions are common throughout the claystone. Two horizons contain spherical to platy barite nodules (∼2 cm in diameter) in olive-gray claystone. Fossils of land mammals, turtles, fish, crocodiles and gastropods (*Hemisinus* (*Longiverena*) *oeciscus*) are present locally in claystone, as noted by Whitmore and Stewart [23], Woodring [40] and MacFadden [26]. Four lignite beds are present in the upper half of the Cucaracha Formation (Section 8; Figure 9). The Cucaracha Formation contains a distinctive bed of welded tuff 4.3 to 7.7 m thick (also known colloquially as the “ash flow” [30], [40]), which is broadly distributed and serves as a useful marker bed (Figure 6).

The Cucaracha Formation represents a coastal delta plain that consists of channel, levee, flood plain and marsh deposits (Figure 9). Abundant paleosols indicate that soils commonly developed on these deposits. Retallack and Kirby [28] recognized 12 different pedotypes that represent as many vegetation types, including mangrove, freshwater swamp, marine-influenced swamp, early successional riparian woodland, colonizing forest, dry tropical forest and woodland. Oxygen and carbon isotopic analyses of land mammal teeth are consistent with these interpretations, as they indicate diverse, C3 plant communities, possibly ranging from dense forest to more open woodland [24]. The pebble conglomerate bed at the base of the Cucaracha Formation represents a fluvial-channel deposit that is broadly distributed (based on its geometry and sedimentology, which are typical of fluvial-channel deposits [62], [63]). Incision of this channel into underlying marine mudstone and sandstone of the Culebra Formation indicates that part of the underlying section has been eroded by the channel. The pebble conglomerate contains fragments of wood that show no evidence of teredinid borings (unlike the wood found in the underlying Culebra Formation), suggesting that this basal conglomerate was deposited above sea level. The presence of oyster fragments probably represents reworking of the underlying marine Culebra Formation. Interbedded lenses of pebble conglomerate and lithic wacke further upsection represent small fluvial channels, based on their lenticular geometry and sedimentology [59], [62], [63]. The small ratio of channel deposits to claystone (the sandstone/claystone ratio for the entire formation is 18.4%) suggests that these were small meandering channels (there is generally a good correlation between channel pattern and sediment load, such that the sandstone/shale ratio provides a clear view of stream type, where meandering channels have relatively low ratios and braided channels have high ratios [59]). Thick sequences of claystone represent flood-basin deposits on the coastal delta plain. Most intervals of claystone show some evidence of soil development [28]. Evidence for paleosols include horizons of calcite and barite nodules, rhizoconcretions, drab-haloed root traces, mottling, relict bedding, gradational contacts between soil horizons C, B and A, and abrupt contacts between soil horizon A and overlying sediment (criteria of [64]). Paleosols indicate periods of stability in between fluvial events of thousands to tens of thousands of years when soils developed on flood-basin or channel deposits [64]. The four lignite interbeds represent histosols of tidal or poorly drained distributaries that penetrated the coastal delta plain, where thick vegetation resulted in the accumulation of much organic matter into layers of peat within marshes [65]. The single interbed of welded tuff represents a pyroclastic, ash-flow deposit (ignimbrite) produced by a nearby explosive eruption. Conformably overlying the Cucaracha Formation is a basalt flow of the Pedro Miguel Formation (Section 8). Underlying claystone in the Cucaracha Formation shows baking and the overlying basalt shows hydrothermal alteration.

#### Land Mammal Biostratigraphy

We found fossils of land mammals throughout the Cucaracha Formation. Land mammal fossils of the peccary cf. *Cynora* sp., the artiodactyl *Paratoceras wardi*, the oreodont *Merycochoerus matthewi* and the rhinoceroses *Menoceras barbouri* and *Floridaceras whitei* were found in Sections 6, 7 and 8 (Figure 6). Taken together, the age of these land mammals indicates a latest Arikareean to middle Barstovian age (19.5 to 14 Ma), with a middle Hemingfordian age (18 to 17 Ma) likely (Figure 8).

## Discussion

**Figure 10:**
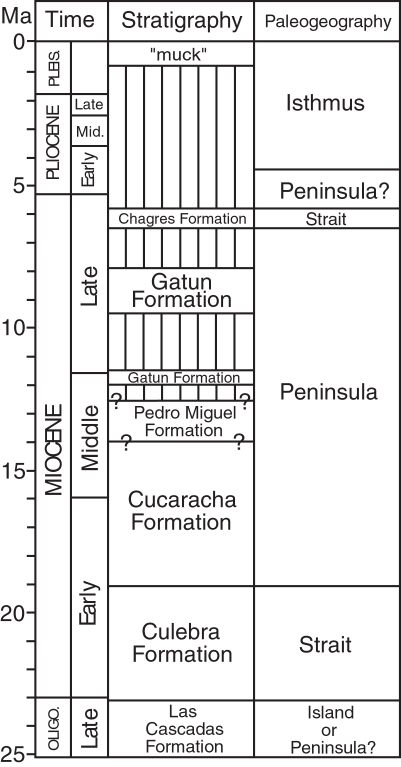
Geologic history of the Panama Canal Basin, showing geologic time, stratigraphy and paleogeography. Vertical lines represent a hiatus. Question marks under stratigraphy represent uncertainty in age. Time scale from Gradstein et al. [69]. Oligo. = Oligocene. Pleis. = Pleistocene.

We infer that the Central American Peninsula was not short-lived in the early Miocene, based on our revised stratigraphy for the Gaillard Cut (Figure 10). We instead find no evidence for the disruption of this peninsula until 6 Ma, when there is evidence for a short-lived strait across the Panama Canal Basin [22]. This conclusion is different from that of Whitmore and Stewart [23], who were the first to present evidence that Panama “was connected to North America by a land area of considerable size and stability” in the middle Miocene, based on the land mammal fossils from the Cucaracha Formation (p. 184). They also concluded, however, that the presence of the Culebra and La Boca formations indicated inundation by the sea both before and after, respectively, the time when the land mammals arrived to Panama by land from North America [23]. Returning the La Boca Formation to the lower part of the Culebra Formation removes the evidence for a transgression after deposition of the Cucaracha Formation.

**Figure 11:**
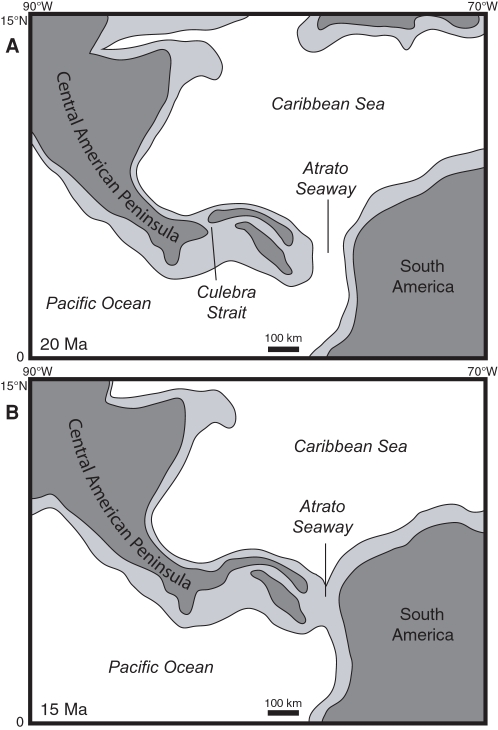
Paleogeographic reconstructions of Central America for (A) 20 Ma and (B) 15 Ma. Light gray represents the outline of tectonic plates containing continental or volcanic-arc crust. Dark gray represents subaerial land. Base maps for the reconstructions were derived from the ODSN Plate Tectonic Reconstruction Service (http://www.odsn.de/odsn/services/paleomap/paleomap.html). The location of subaerial land is based on this study and the distribution of Cretaceous to Tertiary continental and volcanic terranes as derived from Case and Holcombe [70].

The earliest evidence for a terrestrial connection to North America is 19 Ma, based on an estimated age of 19.12–19.83 Ma of the pectinid bivalves found 2 m below the land mammal fossils in the upper Culebra Formation (Section 3 in Figure 6). Given that the upper part of the Cucaracha Formation may be as young as 14 Ma, and that there are at least 355 meters of undated, terrestrial, volcanic rocks of the Pedro Miguel Formation overlying the Cucaracha Formation, we think it likely that the peninsula existed in this part of Central America for much of the Miocene. Low precipitation and temperature estimates derived from the paleosols of the Cucaracha Formation imply a rain shadow from a very high volcanic mountain range (1400–4000 m), suggesting that this peninsula had very high relief [28]. Shallowing to outer neritic depths in the northerly peripheral Limon and Bocas del Toro basins [3], [4] (Figure 1) is also consistent with the continuing emergence of a Central American Peninsula to the south of these basins by the middle Miocene. In addition, the stratigraphically higher Gatun Formation, which is exposed in the northern part of the Panama Canal Basin, contains fossil benthic foraminifera that have a strong Caribbean affinity, indicating an effective biogeographic barrier between Caribbean and Pacific surface water in the middle to late Miocene [22], which further suggests that a peninsula existed during this time. Although we do not have evidence for a direct land connection between Panama and North America after deposition of the Cucaracha Formation, we think it likely that once a peninsula had formed, it would be more probable for its continued existence as a peninsula than for its reversion back to an archipelago. Based on all the above evidence, we think it unlikely that southern Central America existed as a complex island-arc archipelago after the early Miocene, as suggested by Coates et al. [2], Coates and Obando [5] and Collins et al. [22]. The evidence presented here indicates that the main axis of the volcanic arc in southern Central America had coalesced into a subaerial peninsula connected to North America by 19 Ma (Figure 11).

Nevertheless, geologically ephemeral straits across the Central American Peninsula did exist intermittently during the Neogene, as evidenced by the short-lived strait across the Panama Canal Basin 6 Ma [22]. In addition, bathyal sediments in the upper member of the Culebra Formation suggest that a short-lived strait may have existed across the Panama Canal Basin between 21 and 20 Ma (Figure 11A). Other ephemeral straits may have existed intermittently across the San Carlos basin in northern Costa Rica and southern Nicaragua [22], [66]. However, these short-lived straits probably had little impact on the long-term evolution of the marine and terrestrial biota of the Central American Peninsula [22]. Of course, the Central American Seaway (also called the Atrato Seaway), located between Central and South America, remained open until the final formation of the Isthmus of Panama by 3 Ma [1]–[5]. The lack of any South American land mammals in the Cucaracha Formation indicates that such a seaway must have existed in the early to middle Miocene [23], [26]. The Central American Seaway was, therefore, the ultimate barrier to the migration of North American land mammals into South America, not the ephemeral straits that may have formed intermittently across the Central American Peninsula through the Neogene.

The transgressive-regressive facies pattern recorded in the Culebra Formation cannot be easily correlated with the major sea-level fluctuations of the early Miocene. For example, the transgressive-regressive pattern observed in the Culebra Formation appears to conflict with the global sea-level curve of Haq et al. [67]. The lower member of the Culebra Formation, dated between 23 and 21 Ma, indicates a local transgression during this interval, which is opposite from their global sea-level curve, which shows a lowering of sea level during this interval [67]. The upper member of the Culebra Formation, dated between 21 and 19 Ma, indicates a regression during this interval, which is opposite from the sea-level curve, which shows a rising of sea level during this interval [67]. The transgressive-regressive facies pattern in the Culebra Formation also appears to conflict with the global sea-level curve of Miller et al. [68], which shows sea-level fluctuations of 20 m or less between 23 and 19 Ma. These fluctuations are much too small to account for the transgressive-regressive facies pattern observed in the Culebra Formation. The simplest explanation for these discrepancies is that subsidence followed by uplift resulting from regional tectonic forces had a much larger effect on relative sea level within the Panama Canal Basin than did eustasy between 23 and 19 Ma.

The existence of a Central American Peninsula containing a high volcanic mountain range for much of the Miocene has profound implications for our understanding of the tectonic, climatic, oceanographic and biogeographic history related to the formation of the Isthmus of Panama. A Central American Peninsula during the Miocene implies that: (1) uplift of the main axis of the Central American volcanic arc had already occurred near the beginning of the Neogene, (2) the changes in paleogeography thought to be responsible for intensification of the Gulf Stream and down-welling in the north Atlantic occurred much earlier than the Pliocene, (3) terrestrial communities between North and Central America were much better connected in the early to mid-Neogene than previously thought and (4) ocean circulation and biogeographic connection between the Pacific and Caribbean had to have been much more constricted in the early Neogene than previously thought.

### Conclusions

Lithostratigraphic, biostratigraphic and Sr chemostratigraphic analyses demonstrate for the first time that the main axis of the volcanic arc in southern Central America more than likely existed as a peninsula connected to northern Central America and North America for much of the Miocene. The Culebra Formation dates from 23 to 19 Ma, with the Emperador Limestone dating from 21 Ma. The overlying Cucaracha Formation dates from 19 to possibly 14 Ma. What has been called the La Boca Formation underlies, not overlies, the Cucaracha Formation. We, therefore, re-interpret the La Boca Formation (with the Emperador Limestone) as the lower part of the Culebra Formation, as originally proposed by Woodring and Thompson [30].

Our revised stratigraphy for the Gaillard Cut shows that the Culebra Formation represents a transgressive-regressive, marine sequence with environments that include, from lowermost to uppermost: lagoon, fringing reef, neritic, upper bathyal and prograding delta. Bathyal sediments in the upper member of the Culebra Formation suggest that a short-lived strait may have existed across the Panama Canal Basin sometime between 21 and 19 Ma. The overlying Cucaracha Formation represents a coastal delta plain with environments that include fluvial channel, overbank, floodplain and distributary channel marsh, all with extensive development of paleosols representing mangrove, swamp, woodland and dry tropical forest vegetation types. Both the uppermost Culebra and Cucaracha formations contain fossil land mammals that are Hemingfordian to Barstovian in age (19.5 to 14 Ma).

The earliest evidence for a terrestrial connection between Panama and North America is 19 Ma, based on fossil land mammals with only North American affinities and Sr analyses of fossil corals and bivalves. Our revised stratigraphy for the Gaillard Cut demonstrates that the Central American Peninsula was not short-lived in the early Miocene. We instead find no evidence for the disruption of this peninsula until 6 Ma, when there is evidence for a short-lived strait across the Panama Canal Basin. The existence of a peninsula for much of the Miocene has profound implications for our understanding of the tectonic, climatic, oceanographic and biogeographic history related to the formation of the Isthmus of Panama.
